# Laparoscopic vs. open colectomy for T4 colon cancer: A meta-analysis and trial sequential analysis of prospective observational studies

**DOI:** 10.3389/fsurg.2022.1006717

**Published:** 2022-11-01

**Authors:** Peng Chen, Hang Zhou, Chuwen Chen, Xin Qian, Lie Yang, Zongguang Zhou

**Affiliations:** ^1^ Department of Gastrointestinal Surgery, West China Hospital, Sichuan University, Chengdu, China; ^2^ Institute of Digestive Surgery, State Key Laboratory of Biotherapy and Cancer Center, West China Hospital, Sichuan University, Chengdu, China; ^3^ Department of Vascular Surgery, West China Hospital, Sichuan University, Chengdu, China; ^4^ Department of Vascular Surgery, The Affiliated Hospital of Southwest Medical University, Luzhou, China

**Keywords:** laparoscopic colectomy, open colectomy, T4 colon cancer, survival, meta-analysis

## Abstract

**Background:**

To evaluate short- and long-term outcomes of laparoscopic colectomy (LC) vs. open colectomy (OC) in patients with T4 colon cancer.

**Methods:**

Three authors independently searched PubMed, Web of Science, Embase, Cochrane Library, and Clinicaltrials.gov for articles before June 3, 2022 to compare the clinical outcomes of T4 colon cancer patients undergoing LC or OC.

**Results:**

This meta-analysis included 7 articles with 1,635 cases. Compared with OC, LC had lesser blood loss, lesser perioperative transfusion, lesser complications, lesser wound infection, and shorter length of hospital stay. Moreover, there was no significant difference between the two groups in terms of 5-year overall survival (5y OS), and 5-year disease-free survival (5y DFS), R0 resection rate, positive resection margin, lymph nodes harvested ≥12, and recurrence. Trial Sequential Analysis (TSA) results suggested that the potential advantages of LC on perioperative transfusion and the comparable oncological outcomes in terms of 5y OS, 5y DFS, lymph nodes harvested ≥12, and R0 resection rate was reliable and no need of further study.

**Conclusions:**

Laparoscopic surgery is safe and feasible in T4 colon cancer in terms of short- and long-term outcomes. TSA results suggested that future studies were not required to evaluate the 5y OS, 5y DFS, R0 resection rate, positive resection margin status, lymph nodes harvested ≥12 and perioperative transfusion differences between LC and OC.

**Systematic Review Registration:** https://www.crd.york.ac.uk/PROSPERO/, identifier: CRD42022297792.

## Introduction

Colorectal cancer is both the third most commonly diagnosed cancer and the third cause of cancer-related death globally (1). In addition, colon cancers account for nearly 60%, while approximately 106,180 new cases will be confirmed in 2022 (1, 2). Among them, about 15% of colon cancer patients diagnosed with locally advanced disease (T4 stage) (3). Compared with open colectomy, the widely used minimally invasive surgical technology for colon cancer has better short-term results and comparable tumor prognosis (4–8). Moreover, based on the several large randomized controlled trials such as COLOR, CLASICC, COST, EnROL trial and several recent meta-analyses, the NCCN guidelines for colon cancer (2006) recommended that minimally invasive colectomy was considered for colon cancer and performed only by surgeons experienced in this techniques (9–20). However, since the tumor volume of T4 colorectal cancer is large and invades surrounding tissues or adjacent organs, laparoscopic (Lap) En-bloc resection is difficult and risky. Several large randomized controlled trials have compared laparoscopic and open colectomy. But in the Barcelona, ALCCaS, COST, COLOR, MRC CLASSICC, ACOSOG Z6051 trials, locally advanced colon tumors were portion of the exclusion criteria (20–25). Later, most clinical studies enrolled fewer cases of T4 colorectal cancer (20, 25–27). Therefore, there is limited evidence-based data to prove the safety and effectiveness of laparoscopic resection for T4 colon cancer. Laparoscopic T4 colorectal cancer resection is considered to be a technique demanding accuracy and its efficacy is still controversial. The American Joint Committee on Cancer (AJCC) TNM staging system and guidelines from the European Association of Endoscopic Surgery (EAES) guidelines did not recommend laparoscopic surgery for T4 colon cancer (28). However, due to the innovation and progress of laparoscopic platform and the popularization and improvement of laparoscopic technology, surgeons in some large and well-experienced centers tried to apply laparoscopic technology in T4 colorectal cancer and achieved better short-term benefits and oncological outcomes similar to open surgery (4, 6, 8, 29, 30).

Recently, three updated meta-analyses showed that LC was associated with better perioperative outcomes like a lower complication compared with OC and R0 resection rates, 5y OS, and 5y DFS for OC and LC were similar (4, 6, 8). Nevertheless, most of the cases included in the above three meta-analyses were retrospective studies, and the huge heterogeneity caused by different definitions of T4 (T4a vs. T4b, clinical T4 vs. pathological T4). In addition, with more and more statistical analysis of the accumulated literature, the possibility of observing false negative or false positive results increased (31). Trial Sequential Analysis (TSA) can overcome the above shortcomings (32, 33). Therefore, we used TSA method in meta-analysis to control the risk of type I errors.

In this study, we systematically reviewed the existing relevant literature and performed a meta-analysis comprised of TSA of the data on short- and long-term outcomes of LC vs. OC for pT4 colon cancer.

## Methods

This meta-analysis was carried out according to the PRISMA (Preferred Reporting Items for Systematic Reviews and Meta-Analyses) and AMSTAR (Assessing the methodological quality of systematic reviews) Guidelines (34). Ethical consent was not applicable. The present study was registered in PROSPERO website (https://www.crd.york.ac.uk/PROSPERO/) and the Registration Number is: CRD42022297792.

### Literature search

A systematic literature search was carried out in the PubMed, Web of Science, Embase, Cochrane Library, and Clinicaltrials.gov from inception to June 3, 2022 with no limit. The main terms were: (Colonic Neoplasms OR Colonic Neoplasm OR Colon Neoplasm OR Colon Cancer OR Colonic Cancer OR Colonic malignancy OR Colon tumor OR Colon tumour OR colon carcinoma) AND (Locally advanced OR T4 OR multivisceral OR advanced OR pT4 OR cT4) AND (Laparoscopy OR laparoscopic OR open OR minimally invasive OR minimal invasive). In addition, a manual search of references of relevant literatures and reviews was also conducted obtain more potential research.

### Inclusion and exclusion criteria

The inclusion criteria for the meta-analysis were: (1) studies with patients with primary colon cancer; (2) clinical studies that compared LC vs. OC; and (3) raw data that included followings: conversion rate, postoperative complications, perioperative transfusion, mortality, survival, R0 resection rate, resection margin status, number of harvested lymph nodes, and recurrence. The exclusion criteria were: (1) studies did not present data of T4 tumors, (2) mix with rectal cancer or other T stage, (3) studies with no comparison cohort, (4) reviews or meta-analyses, (5) conference abstract, (6) letter, (7) study could not be retrieved.

### Study selection and quality assessment

Three authors (PC, HZ and CC) independently used the Methodological Index for Non-Randomized Studies (MINORS) instrument to assess the quality of the included prospective observational studies (35). The items were scored 0 (not reported), 1(reported but not enough) or 2 (reported and enough). The full score of non- comparative research is 16 points, and the total score of comparative research is 24 points. Moreover, the Grading of Recommendations Assessment, Development, and Evaluation system (GRADE system) was used to rate the level of evidence as very low, low, moderate, or high and created a summary table with the GRADE profiler software (version 3.6.1) (36). Any differences were resolved through consensus discussion between the review group.

### Data extraction

Three researchers (PC, HZ and CC) used structured tables to extract data from each study and input the data into the database. The extracted items contained: author, publication year, study period, country, Single or multicenter study, sample size, gender, age, body mass index(BMI), American Society of Anesthesiologists (ASA), Tumor, Node, Metastasis (TNM) staging classification, neoadjuvant therapy, adjuvant chemotherapy, inclusion and exclusion criteria, median follow-up, conversion rate, operation time (min), blood loss (ml), length of hospital stay (days), soft diet start (days), complications, wound infection, intra-abdominal abscess, ileus, anastomotic leakage, perioperative transfusion, diverting stoma, mortality rate, 5y OS, 5y DFS, R0 resection rate, positive resection margin, lymph nodes harvested ≥12, recurrence.

### Follow-up plans

The follow-up plans were similar in the 3 studies that evaluated long-term results. Patients were followed up at 3 monthly intervals for the first 2 years and every 6 months thereafter. Physical examination and carcinoembryonic antigen (CEA) were routinely performed, whereas abdominal and chest CT scans were performed with an average interval of 6 months. Colonoscopy was carried out once a year or when abnormalities were detected during any follow-up visit. An 18-FDG PET scan was performed if recurrence was suspected. Biopsies were selectively performed.

### Statistical analysis

Analysis was performed using Review Manager (version 5.4.1). Meta-analysis was conducted in which two or more studies assessed the same risk factor in a comparable manner (37). The inverse-variance method and the Mantel-Haenszel estimator were used to calculate pooled mean difference (MD) values and odds ratios (ORs), respectively. MD and pooled ORs were used for continuous variables and dichotomous variables respectively. For continuous variables, if the study only provides median and range values or means and range values, the method described in the previous study was used to calculate the means and standard deviations (38). For the survival endpoints, relative risk (RR) with the corresponding 95% CIs were applied. Statistical heterogeneity was assessed using the Higgins *I*^2^value (39).

The thresholds of low, medium and high heterogeneity(*I*^2^) are 25%, 50% and 75%, respectively. A random-effects model was used for all outcomes (40). Publication bias was evaluated through the funnel plots in Review Manager. *P* < 0.05 was considered statistically significant.

### Trial sequential analysis

TSA was used to evaluate the statistical reliability of data in the cumulative meta-analysis. It controlled the *α* and *β* Value for repetitive testing on the accumulating data. TSA was a tool to assess whether the currently available evidence is sufficiently conclusive (41). Meta-analysis of small samples may increase the risk of false-positive results, resulting in wrong conclusions. To avoid false-negative/positive results, we performed a TSA using the TSA software (version 0.9.5.10, Copenhagen Trial Unit, Denmark). TSA was performed for both dichotomous and continuous outcomes, in which a 20% relative risk reduction, a low-risk-based MD, a type I error (*α* = 0.05, two-sided), and a type II error (*β *= 0.20, power of 80%) were applied to calculate optimal information size.

## Results

### Selected studies and baseline characteristics

**Figure 1:**
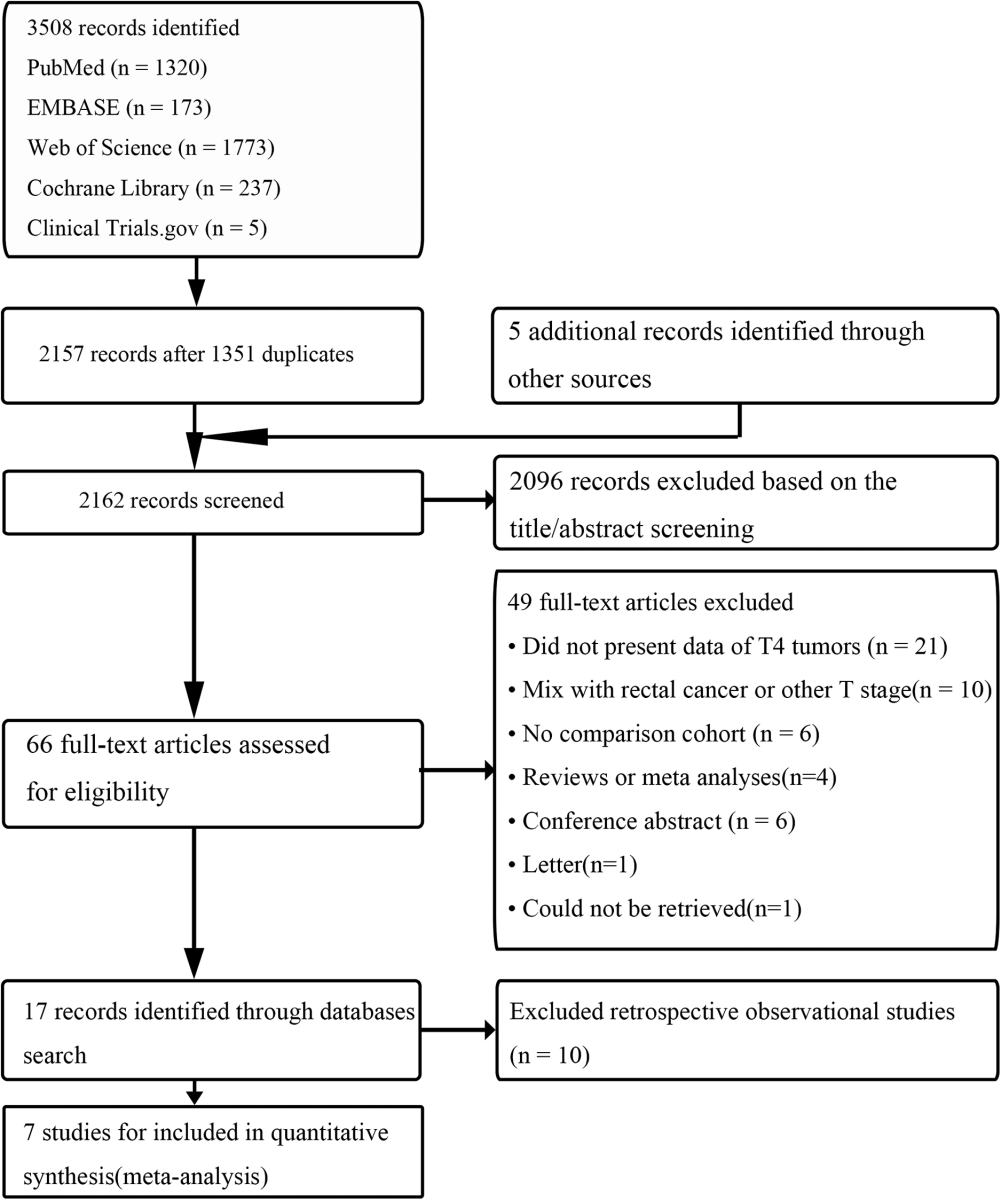
The literature search and selection.

According to the literature search and selection strategy, a total of 7 prospective observational studies that included 1,635 cases with pT4 colon cancer resection (863 LC and 772 OC) were enrolled in this meta-analysis (Figure 1) (42–48). Demographic and clinical characteristics of 7 prospective observational studies were shown in Table 1. Quality assessment of studies was shown in Table 2; 7 studies had a score of >18 points based on MINORS. Meta-analysis for LC vs. OC was shown in Table 3.

**Table 1:** Basic characteristics of included studies.

Reference	Study period	Location	Single or multicenter	n (laparoscopic/open)	Male/female	Mean age (yr)	BMI (mean)	ASA ≥ 3	Tumor stage	T4a/T4b	N + -stage	M1-stage	Neoadjuvant therapy	Adjuvant chemotherapy	Inclusion and exclusion criteria	Median follow-up	MINORS
Bellio 2017	Laparoscopic	2004–2015	Italy	Single	39/38	25/14	73	NR	14/39	II 13/39	38/1	25 (64.1%)	0	NR	11 (28.2%)	Included pT4b	>3 yr	18
										III26/39								
										IV0								
	Open					18/20	77	NR	18/38	II 17/38	29/9	21 (55.3%)	0	NR	17 (44.7)			
										III21/38								
										IV0								
Chan 2016	Laparoscopic	2008–2014	Singapore	Single	93/59	NR	NR	NR	NR	NR	NR	54 (58%)	NS	NR	NR	Excluded emergency cases; direct invasion to adjacent organs or were already metastatic at presentation were also excluded	6.1 yrb	18
						NR	NR	NR	NR	NR		46 (78%)						
	Open																	
de’Angelis 2016	Laparoscopic	2005–2014	France, Switzerland	Multi	106/106	51/55	70.5^b^	24	22/106	II 45/106	pT4a: 91 (86%) pT4b: 15 (14%)	61 (58%)	0	NR	63 (59%)	Excluded emergency setting and those with distant metastasis or synchronous colon cancer	5 yrc	21
										III 61/106								
										IV 0								
	Open					47/59	75.5^b^	25.2	30/106	II 40/106	pT4a: 86 (81%) pT4b: 20 (19%)	66 (62%)	0		53 (50%)			
										III 66/106								
										IV 0								
Elnahas 2015	Laparoscopic	2011–2012	International	Multi	455/406	215/240	67^b^	28	27/428	NR	T4a only	296 (69%)	122 (28%)	20 (4.6%)	NR	Excluded pT4b cancer and rectal cancer	30 d	18
	Open					196/210	67^b^	28	28/378	NR		268 (69%)	111 (28%)	19 (4.7%)				
Kang 2016	Laparoscopic	2003–2013	South Korea	Single	52/57	31/21	61.9^b^	23.4	8/52	II 17/52	pT4a: 45 (87%) pT4b: 7 (14%)	35 (67%)	0	NR	42 (81%)	M1; Robotic surgery	3.4/3.7 yr	20
										III 35/52								
										IV 0								
	Open					39/19	65.4^b^	23.1	3/57	II 24/57	pT4a: 39 (69%) pT4b: 18 (32%)	33 (58%)	0		43 (75%)			
										III 33/57								
										IV 0								
Takahashi 2017	Laparoscopic	2005–2014	Japan	Single	48/36	29/19	68.5	20.6	NR	II 21/48	pT4b: 22 (46%)	14 (29%)	13 (27%)	6.30%	10 (71% of stage III)	Rectal cancer and recurrent cancer were excluded	4.3 yrb	18
III14/48
IV13/48
Open	20/16	71.5	19.6	NR	II 21/36	pT4b: 20 (56%)	7 (19%)	8 (22%)	25%	4 (57% of stage III)
III7/36
IV8/36
Vignali 2013	Laparoscopic	2002–2012	Italy	Single	70/70	37/33	65.2	26.3	1.94 (mean)	II 33/70	pT4a: 52 (74%) pT4b: 18 (26%)	32 (46%)	5 (7.1%)	0 (0%)	52 (74%)	Included pT4 cancer, Excluded rectal and emergency resections	5 yr	19
										III 32/70								
										IV 5/70								
	Open					40/30	63.5	25.8	1.94 (mean)	II 33/70	pT4a: 46 (66%) pT4b: 24 (34%)	32 (46%)	5 (7.1%)	0 (0%)	56 (80%)			
										III 32/70								
										IV 5/70								

*n *= number of patients; NR = not reported.

^a^ Included 17 hand-assisted laparoscopic procedures.

^b^ Median.

**Table 2:** Methodological quality assessment based on MINORS.

Refs	Aim^a^	Inclusion^b^	Prospective^c^	End points^d^	Unbiased^e^	Follow-up^f^	Lost to follow-up^g^	Size^h^	Control^i^	Contemporary^j^	Baseline^k^	Statistical analyses^l^	Total
Bellio (20)	2	2	2	2	1	NA	0	1	2	2	2	2	18
Chan (19)	2	2	2	2	1	2	0	1	2	2	0	2	18
de’Angelis (17)	2	2	2	2	1	2	0	2	2	2	2	2	21
Elnahas (25)	2	2	1	2	1	NA	0	2	2	2	2	2	18
Kang (18)	2	2	2	2	1	2	0	1	2	2	2	2	20
Takahashi (21)	2	2	1	2	1	1	0	1	2	2	2	2	18
Vignali (14)	2	2	1	2	1	2	0	1	2	2	2	2	19

MINORS = methodological index for nonrandomized studies; NA = not applicable.

The following items are scored 0–2 (0: not reported, 1: reported but inadequate, 2: reported and adequate).

^a^ A clearly stated aim.

^b^ Inclusion of consecutive patients.

^c^ Prospective collection of data.

^d^ End points appropriate to the aim of the study.

^e^ Unbiased assessment of the study end point.

^f^ Follow-up period appropriate to the aim of the study

^g^ Lost to follow-up <5%.

^h^ Prospective calculation of the study size.

^i^ An adequate control group.

^j^ Contemporary groups.

^k^ Baseline equivalence of groups.

^l^ Adequate statistical analyses.

**Table 3:** Meta-analysis for LC vs. OC.

Outcome and trials (number of studies)	No. of studies	Sample size	Events	Pooled OR or MD (95% CI)	*I*^2^ (%)	*P* value
Conversion	7	863	95	–	–	–
Continuous variables						
Operation time (min)	5	315/307	–	11.48 [−8.85, 31.81]	58	0.27
Blood loss (ml)	4	276/269	–	−121.12 [−236.08, −6.15]	79	0.04
Length of hospital stay (days)	5	315/307	–	−5.34 [−9.04, −1.64]	76	0.005
Soft diet start (days)	2	158/163	–	−3.58 [−10.14, 2.99]	97	0.29
Dichotomous variables						
Complications	5	315/307	70/109	0.49 [0.31, 0.77]	33	0.002
Wound infection	3	197/201	8/21	0.36 [0.15, 0.86]	0	0.02
Intra-abdominal abscess	2	145/144	10/9	1.08 [0.41, 2.81]	0	0.88
Ileus	3	197/201	15/25	0.41 [0.09, 1.80]	66	0.24
Anastomotic leakage	3	215/214	15/11	1.38 [0.61, 3.12]	0	0.44
Perioperative transfusion	3	631/582	87/137	0.39 [0.28, 0.55]	0	<0.01
Diverting stoma	3	215/214	7/13	0.54 [0.21, 1.41]	0	0.21
Mortality rate	4	276/269	3/2	1.39 [0.26, 7.47]	0	0.7
Oncological outcomes						
5-year overall survival	3	228/233	131/140	0.96 [0.82, 1.12]^a^	0	0.6
5-year disease-free survival	3	228/233	121/125	0.98 [0.81, 1.20]^a^	23	0.85
R0 resection rate	7	863/772	736/665	0.92 [0.69, 1.23]	0	0.57
Positive resection margin	2	561/512	120/100	1.10 [0.81, 1.49]	0	0.53
Lymph nodes harvested ≥12	2	154/142	141/131	0.92 [0.26, 3.25]	48	0.9
Recurrence	2	91/95	28/33	0.84 [0.45, 1.55]	0	0.57

^a^ Pooled RR

### Short-term outcomes

Table 3 showed the results of meta-analysis for all outcomes. All 7 clinical studies reported data on conversion rate with a pooled rate of 11% (95 cases) (42–48). The conversion rate ranged from 7.1 to 28.2% in the LC group. The pooled results showed no significant difference in the operation time between the two groups (MD = 11.48, 95% CI, −8.85 to 31.81, *P* = 0.27). The pooled results showed a significant reduction (MD = −121.12 ml, 95% CI, −236.08 to −6.15, *P* = 0.04) in blood loss among the LC group. LC group showed a significantly lower hospital stay than OC group (MD = −5.34 days, 95% CI, −9.04 to −1.64, *P* =0.005). LC group showed a shorter trend duration than OC group in terms of the number of days to the soft diet start (MD = − 3.58, 95% CI, −10.14 to 2.99, *P* = 0.29).

**Figure 2:**
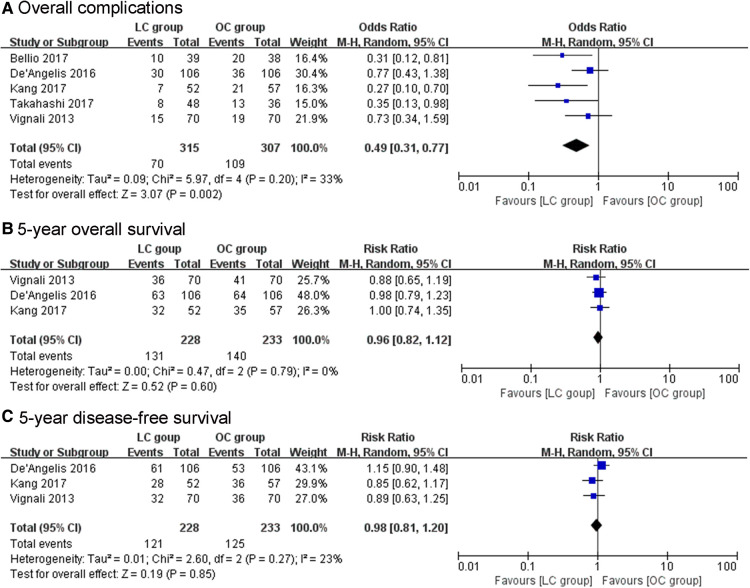
(**A**) The pooled results showed significant decrease in overall complications with LC compared with OC. (**B**, **C**) The pooled results showed no significant difference in 5-year overall survival and 5-year disease-free survival between the treatment groups.

The morbidity rates of LC group ranged from 13.5% to 28.3%, while the morbidity rates of OC group ranged from 27.1% to 52.6%. The overall complications significantly decreased in LC group compared to OC group (OR = 0.49, 95% CI, 0.31–0.77, *P* = 0.002, Figure 2). Among overall complication, the pooled results showed a significant reduction in wound infection among the LC group (OR = 0.36, 95% CI, 0.15–0.86, *P* = 0.02). In addition, the incidence of perioperative transfusion in the LC group was lower than that in the OC group (OR = 0.39, 95% CI, 0.28–0.55, *P* < 0.01). The pooled results showed no significant differences in terms of intra-abdominal abscess (OR = 1.08, 95% CI, 0.41–2.81, *P* = 0.88), ileus (OR = 0.41, 95% CI, 0.09–1.80, *P* = 0.24), anastomotic leakage (OR = 1.38, 95% CI, 0.61–3.12, *P* = 0.44), diverting stoma (OR = 0.54, 95% CI, 0.21–1.41, *P* = 0.21), mortality (OR = 1.39, 95% CI, 0.26 = 7.47, *P* = 0.7), R0 resection rate (OR = 0.92, 95% CI, 0.69–1.23, *P* = 0.57), positive resection margin (OR = 1.10, 95% CI, 0.81–1.49, *P* = 0.53), and lymph nodes harvested ≥12 (OR = 0.92, 95% CI, 0.26–3.25, *P* = 0.9).

### Oncological outcomes

The pooled results showed no significant difference in 5y OS between the LC group and OC group (RR = 0.96, 95% CI, 0.82–1.12, *P* = 0.6, Figure 2). Also, no significant difference was found in the rate of 5y DFS between the groups (RR = 0.98, 95% CI, 0.81–1.20, *P* = 0.85, Figure 2). There was no significant between-group difference in terms of recurrence (OR = 0.84, 95% CI, 0.45–1.55, *P* = 0.57) between the two groups.

### Trial sequential analyses

**Figure 3:**
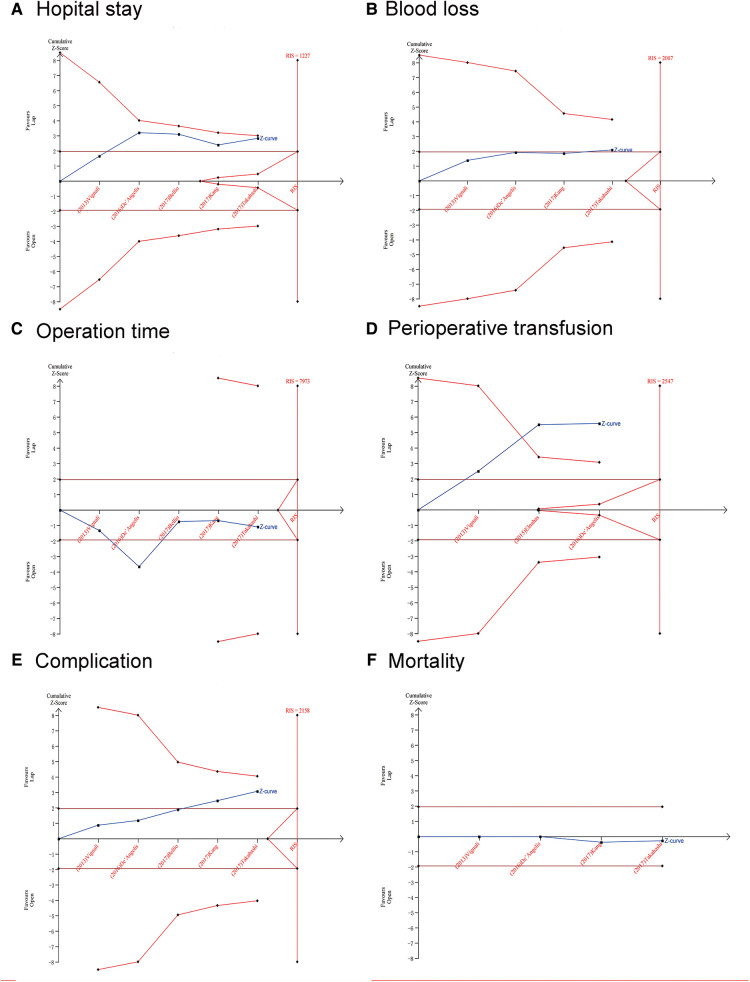
Trial sequential analysis (TSA). The adjusted required information size was calculated using α = 0.05 (two-sided), β = 0.20 (power 80%), and an empirical mean difference. (**A**) Hospital stay (days); (**B**) Blood loss; (**C**) Operation time; (**D**) Perioperative transfusion; (**E**) Complication; (**F**) Mortality.

**Figure 4:**
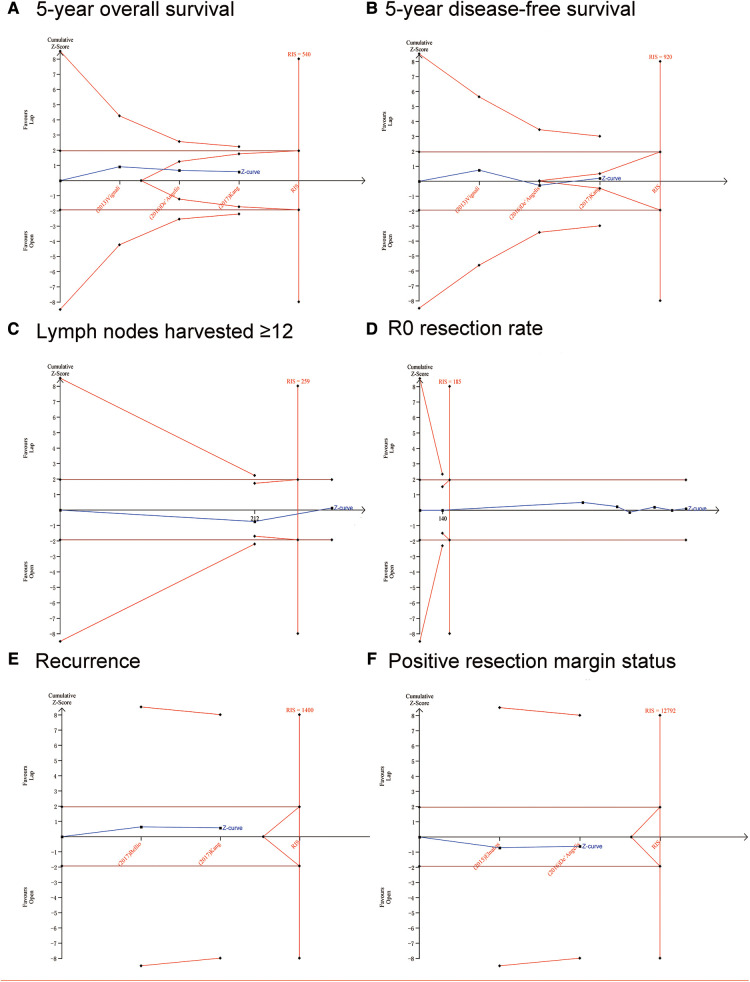
Trial sequential analysis (TSA). The adjusted required information size was calculated using α = 0.05 (two-sided), β = 0.20 (power 80%), and an empirical mean difference. (**A**) 5-year overall survival; (**B**) 5-year disease-free survival; (**C**) Lymph nodes harvested ≥12; (**D**) R0 resection rate; (**E**) Recurrence; (**F**) Positive resection margin status.

**Figure 5:**
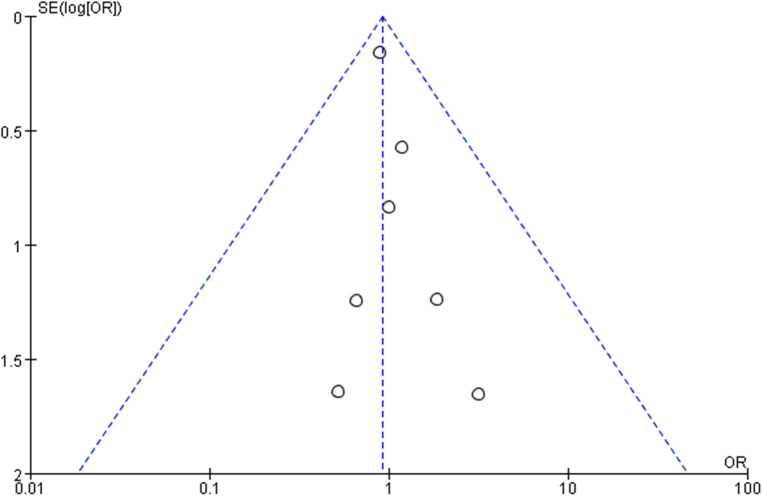
The bilateral symmetry shaped funnel plot of R0 resection rate indicated a lack of publication bias.

The potential false-positive errors of the meta-analysis were found in the length of hospital stay (days) (Figure 3A), blood loss (Figure 3B), operation time (Figure 3C) and complications (Figure 3E), the TSA results showed that the cumulative Z-curve crossed the conventional boundary but did not cross the futility boundaries or the trial sequential monitoring boundary (TSMB). Therefore, more trials are needed before drawing a definite conclusion. For mortality (Figure 3F), recurrence (Figure 4E), and positive resection margin status (Figure 4F), neither the TSMB nor the traditional boundary was crossed, indicating the lack of specific evidence and the need for more research. For perioperative transfusion (Figure 3D), the cumulative Z-curve crossed the TSMB and the traditional boundary, indicating conclusive evidence in the LC group compared with the OC group. The meta-analyses of 5y OS (Figure 4A) and 5y DFS (Figure 4B) did not differ statistically significant; the cumulative Z-curve crossed neither the traditional boundary nor the TSMB, but it crossed the futility boundaries, suggesting no statistical significance between-group difference and no need of further study. The cumulative Z-curve of lymph nodes harvested ≥12 (Figure 4C) and R0 resection rate (Figure 4D) crossed the RIS, suggesting firm evidence of no statistical significance in the LC group compared with OC group.

### GRADE of the outcomes

The GRADE system was applied to synthesize and evaluate the evidence level for the outcomes (Table 4). The power of evidence was moderate in length of hospital stay, complications, ileus, diverting stoma, and wound infection, while it was low in operation time, blood loss, R0 resection rate, mortality, 5y OS, 5y DFS, lymph nodes harvested ≥12, anastomotic leakage, intra-abdominal abscess, infectious complication, recurrence, and adjuvant chemotherapy. The level of evidence was high in perioperative transfusion and very low in soft diet start.

**Table 4:** Strength of evidence for LC in patients with T4 colon cancer compared with OC.

Outcomes	Anticipated absolute effects: Corresponding risk with Lap	95% CI	No of participants (studies)	Quality of evidence (GRADE)
Operation time	The mean in the intervention groups was 11.48 higher		8.85 lower to 31.81 higher	622 (5 studies)	LOW
Blood loss	The mean in the intervention groups was 121.12 lower		236.08 to 6.15 lower	545 (4 studies)	LOW
Length of hospital stay (days)	The mean in the intervention groups was 5.34 lower		9.04 to 1.64 lower	622 (5 studies)	MODERATE
Soft diet start (days)	The mean in the intervention groups was 3.58 lower		10.14 lower to 2.99 higher	321 (2 studies)	VERY LOW
	Study population				
	Corresponding risk with Lap	Assumed risk with Open	Relative effect (95% CI)		
Conversion	120 per 1,000 (101–143)				
R0 resection rate	851 per 1,000 (811–884)	861 per 1,000	OR 0.92 (0.69–1.23)	1,635 (7 studies)	LOW
Complications	212 per 1,000 (146–298)	355 per 1,000	OR 0.49 (0.31–0.77)	622 (5 studies)	MODERATE
Mortality	10 per 1,000 (2–53)	7 per 1,000	OR 1.39 (0.26–7.47)	545 (4 studies)	LOW
5-year OS	575 per 1,000 (483–662)	601 per 1,000	OR 0.9 (0.62–1.3)	461 (3 studies)	LOW
5-year DFS	526 per 1,000 (422–630)	536 per 1,000	OR 0.96 (0.63–1.47)	461 (3 studies)	LOW
Resection margin status	211 per 1,000 (164–266)	195 per 1,000	OR 1.1 (0.81–1.49)	1,073 (4 studies)	LOW
Perioperative transfusion	100 per 1,000 (74–135)	222 per 1,000	OR 0.39 (0.28–0.55)	1,213 (3 studies)	HIGH
Ileus	55 per 1,000 (13–204)	124 per 1,000	OR 0.41 (0.09–1.8)	398 (3 studies)	MODERATE
Lymph nodes harvested ≥12	916 per 1,000 (756–975)	923 per 1,000	OR 0.92 (0.26–3.25)	296 (2 studies)	LOW
Diverting stoma	34 per 1,000 (13–84)	61 per 1,000	OR 0.54 (0.21–1.41)	429 (3 studies)	MODERATE
Anastomotic leakage	70 per 1,000 (32–145)	51 per 1,000	OR 1.38 (0.61–3.12)	429 (3 studies)	LOW
Wound infection	40 per 1,000 (17–91)	104 per 1,000	OR 0.36 (0.15–0.86)	398 (3 studies)	MODERATE
Intra-abdominal abscess	67 per 1,000 (27–158)	62 per 1,000	OR 1.08 (0.41–2.81)	289 (2 studies)	LOW
Infectious complication	130 per 1,000 (85–196)	185 per 1,000	OR 0.66 (0.41–1.08)	538 (4 studies)	LOW
Recurrence	309 per 1,000 (193–452)	347 per 1,000	OR 0.84 (0.45–1.55)	186 (2 studies)	LOW
Adjuvant chemotherapy	619 per 1,000 (498–730)	624 per 1,000	OR 0.98 (0.59–1.63)	538 (4 studies)	LOW

GRADE Working Group grades of evidence.

High quality: further research is very unlikely to change our confidence in the estimate of effect.

Moderate quality: further research is likely to have an important impact on our confidence in the estimate of effect and may change the estimate.

Low quality: further research is very likely to have an important impact on our confidence in the estimate of effect and is likely to change the estimate.

Very low quality: we are very uncertain about the estimate.

### Evaluation of publication bias

A funnel plot of R0 resection rate was applied to visually assess publication bias in this meta-analysis. The funnel plot of R0 resection rate suggested a lack of publication bias (Figure 5).

## Discussion

The safety and oncological outcomes of LC for pT4 colon cancer remain controversial. In the meta-analysis, we included 7 prospective observational studies comparing the efficacy of LC with OC for colon cancer, all of which are scored as high-quality studies based on the MINORS scores (42–48). The results showed that LC could be performed with lesser perioperative transfusion, lesser blood loss, lesser complications, lesser wound infection, and shorter length of hospital stay. Furthermore, there was no significant difference between the two groups in terms of 5y OS, 5y DFS, R0 resection rate, positive resection margin, lymph nodes harvested ≥12, and recurrence. Based on the TSA results, the current evidence for the potential advantages of LC on perioperative transfusion appeared reliable and conclusive. Meanwhile, TSA results suggested that the comparable outcomes in terms of 5y OS, 5y DFS, lymph nodes harvested ≥12, and R0 resection rate drawn from this meta-analysis were reliable and no need of further study.

The perioperative short-term outcomes were significantly more superior in the LC group for colon cancer in terms of lesser blood loss, lesser perioperative transfusion, lesser complications, lesser wound infection, and shorter length of hospital stay. Moreover, the evidence of the advantage of LC on perioperative blood transfusion seemed to be reliable and decisive. Based on the existing literature, LC for T4a colon tumor might be safe, but it should be performed cautiously for T4b colon cancer requiring multivisceral resection (MVR) (6, 49, 50). However, several study groups have reported the safety and effectiveness of Lap MVR (47, 51, 52). Both studies considered that patients with urinary tract invasion were not suitable for lap MVR because the technical complexity and possibility of complications outweighed the gains (47, 48). Nevertheless, with the maturity of laparoscopic surgery technology, especially with the appearance of robotic surgery, ureterectomy and anastomosis had accumulated rich experience. Therefore, this technology depends, at least to some extent, on the technology of urologists in each hospital.

However, several studies reported that MVR was related to high postoperative morbidity and increased risk of conversion (49, 50, 53). Some studies have pointed out that preoperative conversion was associated with poor postoperative outcomes, such as increased postoperative complication rate and mortality, and even a poor prognosis (54, 55). However, studies have reported that conversion has been divided into two types: (I) strategic conversion, which is a prescient decision to avoid complications; (II) reactive conversion, i.e., laparotomy due to unexpected surgical difficulties or complications (56, 57). It is well known that strategic conversion can bring better results than reactive conversion (56). Takahashi et al. reported that except for one case of reactive conversion due to intraoperative bleeding, the other five cases were strategic conversion. The results showed that the reported postoperative complication rate was relatively low, the patient's hospital stay was not prolonged, and the oncological results were favorable. This suggested that strategic conversion might not have a significant unfavorable effect on short- and long-term outcomes.

Another worry is the risk of R1 resection-insufficient clearance of cancer tissue. Our meta-analysis results showed that there were no differences in the R0 resection rate treated with laparoscopic surgery and the evidence of comparable R0 resection rates was reliable. R0 resection was very important for the cancer treatment of T4 patients, and it was also the principal factor affecting the survival after MVR (47, 49, 58). Some people worried that choosing the lap method might threaten the implementation of R0 (48). The COLOR trial, about 20% of T4 patients detected a microscope positive edge (R1), compared to 1% of T3 patients; However, the open group had little superiority (T4, 17.6%; T3, 1.0%) (59). Takahashi et.al reported that the R1 rate in lap group did not increase and two patients in lap group underwent R1 palliative resection for stage IV patients (47). Therefore, the risk of R1 resection in the treatment of T4 tumors with Lap method had not been fully confirmed.

Although the oncological safety of LC in the treatment of colon cancer has been confirmed, there were scarce data on LC in the treatment of T4 colon cancer. In this meta-analysis and TSA of 7 prospective observational studies, there was no significant difference in 5y OS, 5y DFS between two group patients, which was in line with previous studies (4, 60). The above results showed that the oncological results of LC for pT4 colon cancer are acceptable.

Our results also revealed that laparoscopic surgery did not increase the recurrence rate of T4 colon cancer patients when compared with open surgery. Consistent with our research, several large meta-analysis and original research had confirmed this conclusion (4, 8, 61, 62). However, after literature search, there were still several reports that laparoscopic surgery could increase the peritoneal and trocar recurrence rate of T4 colon cancer patients (63–67). Wang et al. reported that laparoscopic colectomy for T4 colon cancer had a higher peritoneal recurrence rate than open surgery (18.1 vs. 10.6 percent; RR 1.56, 1.23–1.99; *P* = 0.0003) (66). In addition, a review published in 1998 showed that trocar recurrence seemed to be secondary to a variety of factors, including pneumoperitoneum, laparoscopic instruments, biologic properties of the tumor, local trauma, and individual surgical skills (63). Therein, careful patient selection in operative approach for T4 colon cancer is needed especially for patients at high risk of intraperitoneal tumor spread.

Recently, there had also been studies on the safety and effectiveness of robot approaches for T4 colon cancer (68–70). An NCDB propensity score-matched analysis of open, laparoscopic, and robotic approaches demonstrated that compared with T4 colon cancer open resection, laparoscopic and robot-assisted surgery had achieved better tumor prognosis and survival rate and robot-assisted surgery was significantly associated with a lower conversion rate compared with laparoscopic surgery (69). This case-matching study demonstrated the safety of using minimally invasive techniques in T4 colon cancers (69). Further multicenter, large-sample randomized controlled trials are needed to verify these results.

Our present meta-analysis has several advantages. The search methodology and inclusion criteria were rigorous, with a systematic literature search to determine the relevant prospective observational studies without restrictions. Further, TSA integrated information indicators and effect indicators, which was more conservative and might be more accurate. In the evaluation setting of non-significant results, TSA could help determine whether “more studies needed” to reduce uncertainty when cumulative Z-curve did not cross the futility boundary. However, this study has some shortcomings. First, there were no randomized controlled trials and no information on quality of life in the literature included in this meta-analysis. Second, because different literatures had different definitions of T4 (T4a vs. T4b, clinical T4 vs. pathological T4), there was heterogeneity between the studies.

## Conclusion

In conclusion, laparoscopic surgery is as acceptable as open surgery for T4 colon cancer in terms of the conversion rate, R0 resection rate, short-term and oncological outcomes. laparoscopic surgery is an innovative and promising approach for the treatment of T4 colon cancer. TSA results demonstrated that further research is not needed to evaluate the 5y OS, 5y DFS, R0 resection rate, positive resection margin status, lymph nodes harvested ≥12 and perioperative transfusion differences between two techniques. Additional multicenter, large-sample randomized controlled trials to evaluate the safety and effectiveness of robot and laparoscope technology for T4 colon cancer are needed in the future.
